# Anthropometric Profile of Latin American Population: Results From the ELANS Study

**DOI:** 10.3389/fnut.2021.740361

**Published:** 2021-11-08

**Authors:** Marianella Herrera-Cuenca, Irina Kovalskys, Alejandro Gerardi, Pablo Hernandez, Yaritza Sifontes, Georgina Gómez, Martha Cecilia Yépez García, Betty Méndez-Pérez, Maritza Landaeta-Jimenez, Rossina Pareja, Lilia Yadira Cortés, Attilio Rigotti, Mauro Fisberg, Iona Zalcman Zimberg

**Affiliations:** ^1^Centro de Estudios del Desarrollo, Universidad Central de Venezuela (CENDES-UCV), Caracas, Venezuela; ^2^Fundación Bengoa para la Alimentación y Nutrición, Caracas, Venezuela; ^3^Facultad de Medicina, Pontificia Universidad Católica de Argentina, Buenos Aires, Argentina; ^4^Escuela de Nutrición y Dietética, Universidad Central de Venezuela, Caracas, Venezuela; ^5^Departamento de Bioquímica, Escuela de Medicina, Universidad de Costa Rica, San José, Costa Rica; ^6^Colegio de Ciencias de la Salud, Universidad San Francisco de Quito, Quito, Ecuador; ^7^Unidad de Investigación en Bioantropología, Actividad Física y Salud, Instituto de Investigaciones Económicas y Sociales Rodolfo Quintero, Universidad Central de Venezuela, Caracas, Venezuela; ^8^Instituto de Investigación Nutricional, Lima, Peru; ^9^Departamento de Nutrición y Bioquímica, Pontificia Universidad Javeriana, Bogotá, Colombia; ^10^Centro de Nutrición Molecular y Enfermedades Crónicas, Departamento de Nutrición, Diabetes y Metabolismo, Escuela de Medicina, Pontificia Universidad Católica, Santiago, Chile; ^11^Instituto Pensi, Fundação José Egydio Setubal, Sabará Hospital Infantil, São Paulo, Brazil; ^12^Departamento de Pediatria, Escola Paulista de Medicina, Universidade Federal de São Paulo, São Paulo, Brazil; ^13^School of Public Health and Preventive Medicine, Monash University, Melbourne, VIC, Australia

**Keywords:** Latin America, ELANS study, obesity, stunting, health risk

## Abstract

**Background:** Latin America has experienced changes in lifestyle since 1960.

**Aim:** The aim was to determine the prevalence of obesity and stunting among eight countries of Latin American and to identify the determinant risk factors for obesity.

**Subjects and Methods:** Data were obtained from 9,218 participants of the Latin American Study of Nutrition and Health (ELANS), a multicenter cross-sectional study of the representative samples in eight Latin American countries. All the participants completed a standard protocol to investigate the nutrient intake and anthropometric variables (weight, height, and circumferences) analyzed by country, gender, age, and socioeconomic status.

**Results:** The prevalence of obesity was higher in Costa Rica and Venezuela (29%) and lower in Colombia (16%), stunting was reported higher in Peru (47%) and lower in Argentina (17%), and waist and neck circumferences showed the higher values in Costa Rica (43%) and Chile (52%) and lower values in Colombia (23 and 26%).

**Conclusion:** This study indicates an increasing trend toward overweight and obesity that are associated with lower socioeconomic status, being a woman, and concurs with inadequate intakes of calcium, which may be related to poor quality diet and in the long term could constitute risk factors for the chronic diseases and a health burden to the region.

## Introduction

Evidence indicates that Latin America (LA) has been experiencing the major changes during the last decades and a relevant one has been the modification of the lifestyle of the population, particularly reflected on the anthropometric characteristics and risks for chronic non-communicable diseases (NCDs) (1).

While the majority of its countries were rural at the end of the 19 and the beginning of the 20 centuries (2), by the 90s decade, a significant proportion of its population had already transitioned into urban life adopting a sedentary lifestyle (3–5).

Jiwani et al. (6) conducted an analysis of the health surveys in 13 Latin American countries between 1998 and 2017 and found an increasing prevalence of obesity in the region, which varied by the wealth and education parameters. In the same study, a reduction in obesity prevalence in Mexico was reported in women, but increased overtime for men. In contrast, in Argentina, obesity prevalence increased among women but remained constant for men (6). These findings show the emergent patterns that parallel existing inequalities within the region and are opportunities to plan the adequate policies.

In the past, the trend toward undernutrition promoted interventions in the region that successfully diminished the rates of malnutrition, most of which had a foundation in the subsidies of foods rich in calories, sugars, and fats (3). However, the 2019 United Nations Food and Agriculture Organization (FAO) report on the State of Food Security and Nutrition in the World that warns about the increasing hunger in the region including hidden hunger associated with obesity (7). Also, an important trend toward stunting in under-five children has been reported with a wide range of variability from 48% in Guatemala to 1.8% in Chile (8). This is a key aspect, since there is evidence of the association between the stunting and obesity later in life (9).

The changes on the lifestyle profile of the Latin American have taken a toll on the health of the population, as stated in the last FAO/Pan American Health Organization (PAHO) report for the LA and Caribbean, showing and confirming a trend toward the overweight and obesity in the region, while still dealing with micronutrient deficits and yet reporting a proportion of undernourished people (10). These phenomena are known as the double burden of malnutrition and hidden hunger (11) and influenced the vulnerability of the Latin American population, mainly associated with growth and development failure in children and increased susceptibility for communicable and NCD (11–14).

The changes in anthropometric characteristics of the LA population deserve a closer look to determine the risks for chronic diseases, diminished quality of life, and human capital in the light of what has been learned in the past decades. Some anthropometric measurements have been key in determining how good is the quality of life and development for societies. For example, stature has been associated with human capital, development, and productivity; waist circumference has been associated with the cardiovascular risks and excess or deficit in body mass index (BMI) with chronic NCD (15). These facts impact the health of the next generations (16–18). Also, neck circumference has been recognized as a marker of upper body subcutaneous adipose tissue distribution, which has been associated with diabetes and cardiovascular disease (19).

In consequence, these facts indicate that adult height is a marker of variation in cumulative net nutrition, biological deprivation, and standard of living conditions between and within population groups. Also, short height has presented an association with obesity in adults (20). As the increased waist, hip, and neck circumferences are risks factors for diseases that overall constitute a deterioration of the quality of life of the population (21), the need for adequate interventions is urgent and designing sustainable changes through the public policies will pay back on the health of the population in the long term.

The primary aim of this study was to determine the prevalence of obesity and stunting in the Latin American population living in the eight countries studied by the Latin American Study of Nutrition and Health (ELANS): Argentina, Brazil, Chile, Colombia, Costa Rica, Ecuador, Peru, and Venezuela and the secondary aim was to analyze the anthropometric indicators according to gender, age group, socioeconomic status (SES), and the adequate intake of iron and calcium and to identify if these factors were associated to a higher risk for obesity.

## Methods

The ELANS is a household-based multicenter cross-sectional study, which aimed to describe the nutritional status in LA and to investigate food and nutrient intake, anthropometric characteristics, and physical activity levels of the representative samples from urban populations within the eight Latin American countries (Argentina, Brazil, Chile, Colombia, Costa Rica, Ecuador, Peru, and Venezuela). The fieldwork for the ELANS study was conducted from 2014 to 2015 (22).

### Sample

The sample includes 9,218 adolescents and adults aged 15–65 years of the eight Latin American countries. Sampling was random multistage, stratified by geographical location, gender, age, and SES only for the urban population. SES was evaluated by using a country-dependent questionnaire format based on the legislative requirements or established local standard layouts and classified in the low, medium, and high levels. Details about sampling are described in a previous publication (22).

### Dietary Assessment

The dietary assessment was performed on a respondent within the household during two independent household visits on non-consecutive days with an interval of up to 8 days between them. Each visit included a 24-h dietary recall conducted by using the Multiple-Pass Method (23) to record in detail all the foods and beverages consumed over the prior day. The 24-h recalls counted on the weekdays and weekend days. The food measures of the household obtained in the 24-h were changed to grams and milliliters by the qualified nutritionists.

The food and beverage intake assessed with the 24-h gave a result: energy, macronutrients, and micronutrient values by using the Nutrition Data System for Research (NDSR) [version 2013 software, University of Minnesota (MN)] after a food matching standardized procedure conducted by the expert nutritionists in each country. The complete food standardization procedure has been described in detail elsewhere (24). The misreporting of energy intake was analyzed and described further in another published paper (25).

#### Usual Intake

Two 24-h were assessed to approximate the usual food consumption and estimate intraindividual variability in nutrient intake. The web-based statistical modeling technique known as the Multiple Source Method (MSM) (https://msm.dife.de/tps/en), proposed by the European Prospective Investigation into Cancer and Nutrition (EPIC), was used to estimate the usual intake of energy and macronutrients. This method was chosen because of its capability for improving estimates of usual dietary intake of energy, nutrients, foods, and food groups by considering within-person variance in the intake, thereby improving the usual intake distribution for the population (23). Due to variations in eating habits among the Latin American populations, the usual intake estimation was performed separately for each country. The relative contribution of each macronutrient to total energy intake was subsequently calculated. After adjusting for misreporting, the plausible reporters were 6,648 individuals (72.1%), who were used for further analyses in this study.

#### Micronutrient Intake Analysis

Based on the dietary intake obtained by the two 24-h recall visits, the intake of iron and calcium was analyzed following the guidelines of the Institute of Medicine estimated required allowances estimated average requirement (EAR) cutoff point method to assess the prevalence of inadequacies, based on which we calculated the Inadequate Micronutrient Intake Index by country. For the purpose of this study, it was decided to use iron and calcium adequacy due to their association with obesity and its associated risks, therefore allowing the analyses of the relationship between BMI and the inadequacy of iron and calcium (22, 26).

### Anthropometry

Trained evaluators measured the anthropometric variables according to the international methodology (27). Each measurement was repeated to ensure accuracy and the average was used for the statistical analyses. A third measurement was taken if the two readings differed by more than the previously established set point. Each country followed the categorization of BMI. Adolescents (15–19 years) were classified based on the cutoff points established by the WHO (28). Sex-specific Z-score BMI-for-age growth charts were used and SD limits were followed accordingly. Underweight was established at BMI for age < −2 SD, normal weight between −2 SD ≤ BMI for age ≤ 1 SD, overweight between 1 SD ≤ BMI for age ≤ 2 SD, and obese BMI for age > 2 SD. The WHO growth charts are regularly used in the Latin American countries; thus, it was selected as appropriate for this and the other ELANS studies (29). For adults (older than 19 years old), BMI was categorized as underweight (<18.5 kg/m^2^), normal weight (18.5–24.9 kg/m^2^), overweight (25–29.9 kg/m^2^), and obese (≥30.0 kg/m^2^) (30).

Waist circumference (WC) classification was based on the reference data by gender, age, and ethnicity for the adolescents compiled by Katzmarzyk et al. (31) and for the adults according to the WHO (30). The waist–hip ratio (WHR) was classified according to the WHO (32) criteria for men >1.0 and women >0.85 as high cardiovascular risks. The neck circumference (NC) for adolescents was categorized as an upper limit if circumference was >34.5 cm for boys and >31.25 cm for girls (33), whereas for adults, the cutoff points for obesity were >39 cm for men and >35 cm for women (19). Cutoff points for defining stunting were 165.5 cm for men and 153.3 cm for women to evaluate the risks of NCD (34).

### Ethics

The ELANS protocol was approved by the Western Institutional Review Board (#20140605) and registered at the Clinical Trials (#NCT02226627). Each country presented the protocol and submitted it for approval at the local ethics committees with signed informed consent for the adolescents and adults. The complete design, protocol, methodology, and standardization of the food composition database utilized in the ELANS study have been documented elsewhere (22, 24).

### Statistical Analysis

Descriptive statistics (with 95 CIs) were calculated by using the central tendency (mean and SD) and distribution statistics (percentiles). The anthropometric and food consumption indicators were analyzed by country, gender, age group, and SES. The sample was weighted by SES and consumption of nutrients was included after misreporting was analyzed and only plausible intakes were considered. Additionally, odds ratio (OR) was used to identify the determinant risk factors of obesity by BMI, adjusted for energy intake, country, and educational level. All the analyses were performed by using the Statistical Package for the Social Sciences (SPSS) software (version 20.0; SPSS Incorporation, Chicago, Illinois, USA). A *p* value equal to or lower than 0.05 was considered as statistically significant.

## Results

The study sample included 9,218 participants of whom 6,648 (72.1%) were considered as plausible reporters; 51.7% women, a mean age of 35.8 years, and 51.6% reported a low SES (these data were presented in the Supplementary Material). Table 1 summarizes the prevalence of different anthropometric indicators according to gender, age group, and SES by country. Women, age group of 50–65 years old, and low SES have the highest prevalence of obesity (BMI), WC, WHR, and stunting with the exception of NC, which was higher in men, 15–19 years old, and high SES. Overall, the highest prevalence of obesity was found in Costa Rica (29.5%) and Venezuela (29.2%); the lowest prevalence was found in Colombia with 16.0%. For WC and NC obesity, the highest prevalence was found in Costa Rica and Chile (43.2 and 41.3%, respectively). In this case, Colombia had the lowest value with 23.4%.

**Table 1:** Comparison of the anthropometric indicators prevalence according to the country of living, gender, age group, and socioeconomic status in the Latin American Study of Nutrition and Health (ELANS)^*^.

**Country**	***n***	**Gender**		**Age group**				**Socio-economic status**			**Total**
		**Female**	**Male**	**15-19**	**20-34**	**35-49**	**50-65**	**High**	**Middle**	**Low**	**Overall**
**Obesity (BMI)**											
Argentina	879	31.9	23.9	7.0	16.7	32.5	47.1	17.3	37.6	46.8	27.7
Brazil	1,406	28.8	21.3	6.8	21.4	29.2	35.7	28.9	48.3	57.0	25.1
Chile	625	33.7	22.0	11.3	21.0	35.0	35.9	18.2	54.7	58.9	27.9
Colombia	875	18.0	13.8	2.5	10.5	22.4	21.3	7.7	40.1	47.5	16.0
Costa Rica	551	35.6	23.5	9.8	21.1	43.8	43.6	38.8	56.7	64.9	29.5
Ecuador	574	31.7	18.2	7.0	16.8	33.7	43.4	23.3	48.8	64.5	24.7
Peru	876	28.6	17.3	5.8	17.9	31.7	34.8	25.7	57.4	57.7	23.1
Venezuela	862	33.3	25.2	12.0	19.8	41.4	41.0	37.2	60.0	51.5	29.2
ELANS	6,648	29.5	20.6	7.7	18.3	32.5	37.1	25.7	48.6	54.6	25.1
**Abdominal obesity (Waist circumference)**											
Argentina	879	45.0	26.5	35.1	16.4	34.9	61.9	20.0	35.0	39.3	35.6
Brazil	1,406	46.8	19.4	33.8	23.5	33.8	52.1	42.2	31.7	33.9	33.7
Chile	625	53.5	28.4	54.8	27.6	45.2	50.6	38.6	36.9	46.0	41.3
Colombia	875	32.7	13.2	26.6	11.7	23.6	40.8	28.2	19.3	26.7	23.4
Costa Rica	551	59.9	25.0	42.2	29.9	50.0	63.7	48.5	39.5	47.1	43.2
Ecuador	574	54.3	15.2	33.7	20.8	41.1	53.5	30.1	28.4	39.3	34.1
Peru	876	55.7	14.9	40.7	23.7	43.6	48.4	34.6	40.5	33.3	35.9
Venezuela	862	53.1	22.6	36.8	22.9	49.8	50.6	47.6	36.3	37.9	38.1
ELANS	6,648	49.0	20.3	37.1	21.8	38.6	52.1	36.8	32.6	36.5	35.0
**Obesity (Neck circumference)**											
Argentina	879	34.3	40.5	54.0	17.5	37.8	54.5	24.3	37.1	40.4	37.5
Brazil	1,406	28.8	29.1	35.6	23.9	29.5	33.8	34.3	29.9	26.8	29.0
Chile	625	50.3	54.7	66.1	37.9	56.5	63.0	50.0	53.1	52.9	52.5
Colombia	875	21.7	30.4	53.2	16.0	29.3	26.1	23.1	23.6	28.1	25.9
Costa Rica	551	39.1	48.5	42.7	28.1	56.6	59.8	44.4	42.8	44.5	43.6
Ecuador	574	29.1	30.4	45.3	15.5	36.8	37.4	28.8	31.3	29.0	29.8
Peru	876	30.8	34.6	44.9	21.7	38.2	39.9	36.3	36.5	28.5	32.6
Venezuela	862	42.5	42.6	48.7	28.4	52.6	49.7	42.9	43.4	42.3	42.5
ELANS	6,648	33.5	37.3	47.1	23.3	39.7	43.9	36.4	35.2	35.3	35.4
**Stunting**											
Argentina	879	20.0	13.3	4.4	16.1	20.4	19.0	14.7	14.1	19.6	16.6
Brazil	1,406	20.3	14.1	3.1	13.5	16.9	32.4	2.2	12.5	25.8	17.3
Chile	625	27.6	18.2	4.8	17.4	22.0	39.6	15.9	20.4	27.9	23.0
Colombia	875	29.1	26.4	10.3	20.7	29.3	43.8	7.5	27.4	29.9	27.8
Costa Rica	551	33.2	22.8	3.7	26.8	35.9	41.2	24.2	24.1	38.7	28.3
Ecuador	574	45.7	41.6	16.3	41.6	52.1	57.6	30.1	41.7	48.3	43.6
Peru	876	47.1	47.0	25.6	42.9	53.1	63.9	30.6	44.8	55.8	47.0
Venezuela	862	22.2	22.6	9.5	18.6	23.3	36.4	18.6	15.4	24.0	22.4
ELANS	6,648	29.1	24.6	9.8	23.7	29.2	39.3	19.2	22.9	31.8	26.9
**Obesity (waist-hip ratio)**											
Argentina	879	39.2	43.1	11.4	20.4	50.7	71.9	32	37.6	46.8	41.2
Brazil	1,406	55.5	48.7	21.9	42.9	56.3	80.0	50	48.3	57	52.2
Chile	625	54.8	57.1	12.9	39.7	66.7	83.1	49.4	54.7	58.9	55.9
Colombia	875	37.7	50.7	16.5	27.5	51.4	70.9	40	40.1	47.5	43.9
Costa Rica	551	62.6	58.9	25.6	48.9	78.6	90.1	66.7	56.7	64.9	60.9
Ecuador	574	60.1	52.7	14.0	44.2	75.5	88.9	45.2	48.8	64.5	56.3
Peru	876	62.5	51.2	14.0	47.3	74.5	85.2	54.6	57.4	57.7	57.0
Venezuela	862	51.0	56.0	8.5	45.2	70.3	75.3	66.7	60.0	51.5	53.5
ELANS	6,648	52.3	51.4	15.7	39.7	63.0	79.1	51.4	48.6	54.6	51.9

* *Values presented include the plausible reporters only (n = 6,648)*.

Stunting was more prevalent in Ecuador and Peru (47 and 43.6%, respectively), whereas Argentina and Brazil were the countries with lower prevalence (16.6 and 17.3%, respectively). The countries with the highest prevalence of increased WHR were Costa Rica and Peru (60.9 and 57%, respectively). On the opposite extreme, Colombia and Argentina reported the lowest prevalence of increased WHR with 43.9 and 41.2%, respectively. For overall anthropometric indicators, the prevalence of obesity and stunting was higher for the 50–65 years group than for those aged 15–19 years both in the women and men. In summary, Costa Rica, Chile, and Venezuela were the countries with more prevalence of obesity by the different indicators, while Colombia showed the lowest prevalence of obesity in general.

Table 2 summarizes the mean and SD of iron and calcium intake according to the demographic indicators studied by country. Sex differences were observed in both the nutrients throughout the eight countries evaluated. The quantities of iron and calcium consumed were higher for men than for women overall; this pattern was consistent across the countries. The largest difference between genders was observed in Costa Rica (3.3 mg/day). In terms of the age groups, people under 34 years old have a higher iron intake in Argentina, Brazil, Colombia, Ecuador, and Venezuela. Iron intake was similar across all the age groups in Chile and Peru. For calcium intake, people between 15 and 19 years old in all the countries, except for Venezuela and Peru, have higher calcium intake than older people. The largest difference between men and women was reported in Argentina (126.4 mg/day).

**Table 2:** Iron and calcium intakes (mg) by country between gender, age group, and socioeconomic status in the Latin American Study of Nutrition and Health (ELANS)^*^.

	***n***	**Gender**					**Age group**									**Socio-economic status**							**Total**	
		**Female**		**Male**		***p***	**15–19 year old**		**20–34 year old**		**35–49 year old**		**50–65 year old**		***p***	**High**		**Middle**		**Low**		***p***	**Overall**	
		**Mean**	**SD**	**Mean**	**SD**		**Mean**	**SD**	**Mean**	**SD**	**Mean**	**SD**	**Mean**	**SD**		**Mean**	**SD**	**Mean**	**SD**	**Mean**	**SD**		**Mean**	**SD**
**Iron intake**																								
Argentina	879	12.7	2.8	15.4	3.2	<0.001	15.0	3.7	14.0	3.1	14.1	3.2	13.6	3.3	0.002	13.8	2.5	14.1	3.4	14.1	3.3	0.196	14.1	3.3
Brazil	1,406	9.2	2.6	11.4	3.2	<0.001	11.0	3.2	10.8	3.3	9.9	2.7	9.3	2.8	<0.001	10.7	3.6	10.3	2.9	10.1	3.1	0.099	10.2	3.1
Chile	625	11.4	3.0	13.9	3.3	<0.001	12.7	3.3	12.8	3.6	12.5	3.4	12.5	3.2	0.795	12.3	3.8	12.4	3.1	13.0	3.5	0.002	12.6	3.4
Colombia	875	14.6	3.0	16.8	3.2	<0.001	15.9	3.0	16.2	3.5	15.6	3.2	14.7	3.0	<0.001	16.0	3.8	15.9	3.1	15.4	3.4	0.018	15.6	3.3
Costa Rica	551	12.4	2.8	15.7	3.7	<0.001	14.3	3.2	14.3	3.7	13.8	3.3	13.4	4.3	0.162	14.4	4.5	14.0	3.3	13.7	3.6	0.158	14.0	3.6
Ecuador	574	13.1	2.5	15.7	2.7	<0.001	14.8	2.8	15.0	2.8	14.2	3.1	13.4	2.6	<0.001	13.9	3.0	14.5	2.8	14.6	3.0	0.533	14.5	2.9
Peru	876	13.2	2.8	15.3	3.5	<0.001	14.1	3.1	14.4	3.4	14.3	3.2	13.8	3.2	0.191	14.3	3.5	14.1	3.1	14.3	3.4	0.650	14.3	3.3
Venezuela	862	12.0	2.7	14.4	3.5	<0.001	13.5	3.4	13.6	3.3	13.1	3.3	12.4	3.4	0.001	13.2	4.6	13.6	3.8	13.1	3.2	0.038	13.2	3.4
**Calcium intake**																								
Argentina	879	679.6	209.3	806.0	256.8	<0.001	774.2	238.1	758.2	232.2	741.8	214.6	710.6	287.5	0.082	817.1	217.2	772.0	267.9	699.6	210.2	<0.001	743.6	242.8
Brazil	1,406	419.5	198.5	475.8	236.4	<0.001	494.9	245.1	465.3	230.3	424.8	200.8	417.8	202.6	<0.001	505.9	229.4	485.0	233.3	391.6	186.8	<0.001	446.4	219.2
Chile	625	488.2	187.9	546.9	207.8	<0.001	578.0	223.7	526.8	191.2	490.7	195.8	508.0	201.7	0.021	531.8	164.2	541.7	210.3	490.0	197.8	0.010	516.8	199.8
Colombia	875	664.7	197.0	714.8	209.3	<0.001	700.5	212.7	701.4	206.5	693.2	209.5	657.2	188.3	0.092	770.6	208.4	722.9	192.1	651.3	207.5	<0.001	688.6	204.4
Costa Rica	551	426.7	172.4	493.5	166.0	<0.001	468.9	189.8	463.8	168.9	445.3	160.4	457.5	183.1	0.715	534.6	192.5	470.3	163.4	387.3	149.4	<0.001	458.6	172.5
Ecuador	574	620.8	168.4	685.6	199.2	<0.001	682.0	202.2	651.5	192.3	647.8	178.2	646.8	179.0	0.519	690.7	206.9	699.7	192.0	612.0	169.4	<0.001	654.2	187.6
Peru	876	440.8	123.8	466.7	137.6	0.004	440.6	124.9	458.6	137.9	452.9	129.8	452.6	123.2	0.635	488.8	145.9	455.9	129.7	436.2	122.3	<0.001	453.4	131.3
Venezuela	862	613.2	208.6	683.3	255.4	<0.001	642.3	225.1	665.5	249.3	625.4	216.5	650.3	240.4	1.395	678.1	229.5	692.5	244.8	637.0	232.9	0.029	647.7	235.4

* *Values presented are means and SD and include the plausible reporters only (n = 6,648)*.

The results did not show a significant association between the SES and iron consumption. There were no significant differences for most countries, except for three countries with statistical differences between the SES groups. The higher iron intake was observed in low SES for Chile, medium SES for Venezuela, and high SES for Colombia. The highest variation, according to the SES, was observed in calcium consumption. In most countries (Argentina, Brazil, Colombia, Costa Rica, and Peru), the highest SES have higher intakes, while in Chile, Ecuador, and Venezuela, the highest consumption was observed in the middle level.

Overall, Brazil and Chile had the lowest iron consumption and the highest averages were observed in Colombia and Ecuador. The difference between Brazil and Colombia overall iron intake was 5.4 mg. For overall calcium intake, the highest values were reported in Argentina and Colombia and the lowest values were reported in Brazil and Peru. The mean difference between Argentina and Brazil overall calcium intake was 297.2 mg.

Table 3 presents the logistic regression analysis results describing the effect of gender, age group, SES, iron intake, and calcium intake on obesity at the regional level. In general, female increases the risk of obesity by 62% (OR = 1.62; 95% CI = 1.45–1.81; *p* < 0.05), particularly in Ecuador where the OR = 2.08 (95% CI = 1.41–3.06; *p* < 0.05). These data were presented in the Supplementary Material.

**Table 3:** Association between the main characteristics of the subject and obesity in the Latin American Study of Nutrition and Health (ELANS).

		**OR**	**95% CI**		***P***
Gender	Male	1.00			
	Female	1.62	1.45	1.81	<0.05
Age group	15–19	1.00			
	20–34	0.55	0.49	0.62	<0.05
	35–49	1.70	1.52	1.91	<0.05
	50–65	2.09	1.84	2.38	<0.05
Socio-economic status	High	1.00			
	Middle	0.88	0.78	0.98	<0.05
	Low	1.12	1.00	1.25	<0.05
Intake	Within the iron recommendation	1.00			
	Under iron recommendation	0.92	0.69	1.22	>0.05
	Within the calcium recommendation	1.00			
	Under calcium recommendation	1.19	1.01	1.41	<0.05

*OR, odds ratio*.

*Regression model by using obesity (BMI) as dependent variable, adjusted for energy intake, country, and educational level*.

Considering the age group, the more the age, the more the risk of obesity. It increases two-fold the risk in the 50–65 years old category (OR = 2.09; 95% CI = 1.84–2.38; *p* < 0.05) compared to the 15–19 years old category; this pattern was the same across all the countries, especially in Argentina where the risk is greater (OR = 3.19; 95% CI = 2.30–4.42; *p* < 0.05) (see Supplementary Material).

With respect to SES, there was a 12% higher risk of obesity in low SES for all the countries compared with the high SES. When countries were considered independently, the differences among SES were observed only for Chile (OR = 1.80; 95% CI = 1.26–2.57; *p* < 0.05) and Argentina (OR =1.44; 95% CI = 1.07–1.93; *p* < 0.05). In all the other countries, the difference was not significant for the low SES compared with the high SES.

For the micronutrient indicators, it was observed that low iron consumption was not a significant risk factor for obesity. However, in the analysis by country, Venezuela was the only one where underconsumption of iron was statistically associated with obesity (OR = 2.82; 95% CI = 1.03–7.87; *p* < 0.05) compared with within the iron recommendation. In addition, low calcium intake constitutes a risk factor for obesity, increasing in 19% the risk (OR = 1.19; 95% CI = 1.01–1.41; *p* < 0.05) compared with within the calcium recommendation. Nevertheless, in the analysis by country, Argentina was the only one in which poor calcium consumption was a risk factor for obesity (OR = 1.79; 95% CI = 1.27–1.52; *p* < 0.05), whereas the other seven countries did not present an association.

## Discussion

This study showed the prevalence of overweight and obesity in the eight Latin American countries related to findings of BMI, NC, WC, and WHR and showed that women had a 62% higher risk of becoming obese compared to men and there was a tendency toward a higher prevalence of obesity in higher SES in some countries. In addition, 26.9% of the total population was stunted and women had a higher prevalence than men (29.1 and 24.6%, respectively) and subjects with calcium consumption below recommendations had 19% more risk of becoming obese than those with calcium consumption within the recommendations. Obesity is a risk factor for chronic NCD; therefore, presenting the best available data and analyzing it in a more harmonized approach is a key to finding cost-effective measures that prevent the diseases and contribute to the health of the population (6).

Diverse factors contribute to the prevalence of obesity: from social determinants of health to environmental exposures in critical periods of growth and behavioral aspects and conducts (35, 36). Other studies from the ELANS studied diet quality and diversity finding that women scored lower when indexes for quality and diversity were evaluated and total diet quality scores were higher as the socioeconomic level increased and unhealthy scores were the opposite (37).

Evidence shows that the ultimate stature and shape of an adult result from a continuous interaction between the genetic and environmental influences during the periods of growth (38, 39). Shorter statures in the adults as reported in Ecuador and Peru might well be evidence of the genetic characteristics of traits of ethnicity present in the Ecuadorian and Peruvian populations, on which the indigenous phenotypes are evident according to the genomic studies (40). However, the anthropometric characteristic of these Latin American populations could have also been influenced by the interactions of the effects of good or poor nutrition, urban or rural populations, at least when the growth period was active, and the effects of altitude or living in the tropics were evident (38).

Henriques et al. (20) report a positive association between the short stature and obesity measures for women in a study of Portuguese adults, as shorter women are more likely to be overweight than their taller counterparts, independently of age.

The stature as a measurement impacted by cumulative wellness or deprived life can show a path for analyzing some characteristics of a population, the quality of life achieved, and ultimately the human capital of the nation (16). In addition, a short height can be associated with an increased risk for mortality and cardiovascular disease events, thus impacting the quality of life of individuals (15). Paajanen et al. (34) conducted a systematic review of the literature and a meta-analysis of short stature and its association with coronary heart disease. They found a combined relative risk for all-cause mortality for short men of 1.37 (1.29–1.46) and short women 1.55 (1.41–1.70). This study also found higher risks for different kinds of cardiovascular endpoints for the shortest individuals compared to the tallest categories (34).

As stature, other anthropometric indicators have proven effective in identifying risk for chronic NCD. Among those, BMI, WC, WHR, and NC have been markers for the risk of NCD such as obesity, diabetes, dyslipidemias, and high blood pressure, among others (41–43).

In this study, the countries showing the highest prevalence of obesity according to BMI were Costa Rica and Venezuela; however, when analyzing other markers showing a more specific distribution of body fat such as NC, WC, and WHR, Chile and Costa Rica show the highest prevalence of elevated WC and NC. With respect to WHR, Costa Rica and Peru show the highest proportion of obesity. Colombia had the lowest prevalence of obesity.

According to the World Heart Federation (WHF) (44), in 2013, Brazil showed an obesity prevalence of 18.8% in the adults. In contrast, this study shows an increase to 25.1% of the obese adults; this rapid increase is taking a toll on NCD, particularly diabetes and cardiovascular diseases.

In 2016, the regional meeting of the WHF (44, 45) showed that the burden of heart conditions in Latin America was high and that raising awareness on the cost-effective prevention strategies was urgently needed. At the time, heart disease affected nearly a third of the population in Brazil (32%), 38% in Chile, 17% in Colombia, 14% in Ecuador, 16% in Peru, and 33% of the population in Venezuela. The WHF study included Mexico, El Salvador, and Panama and did not include Argentina and Costa Rica (45).

Regional and national food consumption patterns among the different SES groups can mask the inequalities by showing nutrition indicators that might not be as accurate to show the real needs of the population. Consequently, translating inequalities in nutrition indicators that show the regional and socio-economic characteristics of the countries is a key to finding the routes to well-being for all in a sustainable way (46). One of the largest gaps in the region is diet quality intake, reported by the ELANS in another study, according to which only 7.2% of the overall sample reached the recommendations of the WHO for the fruits and vegetables consumption, a previously known risk for NCD (47).

As a cost-effective policy for the prevention of NCD includes a healthy diet that can provide the variety of nutrients the body needs for its adequate function, we evaluated the intake of two micronutrients associated with obesity; those were calcium and iron. Calcium has been proposed to have possible antiobesity mechanisms including regulation of adipogenesis, fat metabolism, thermogenesis, fat absorption, and gut microbiota, among others (48, 49). Thus, an inadequate intake of calcium, as shown in this study and other works by the ELANS, can be a potentially disruptive factor in achieving a healthy BMI. On the other hand, iron has been associated with adiposity-related inflammatory processes that might decrease its absorption (50–52). All the countries showed that the risk for obesity was associated with inadequate intake of calcium. However, the same relationship was not observed for iron; only Venezuela showed a risk for obesity when iron deficit intake was present. The lack of association between the iron intake and risk for obesity might be related to iron fortification in foods included in the usual dietary intake of the population and enrichment existing regulations in the countries. In addition, this study found few cases of iron inadequate intake, which might be due to consumption of the fortified foods as previously explained and the 24-h recall might not capture accurately the iron intake, despite the adjustments for misreporting conducted on the ELANS research (25).

It should be mentioned that according to the latest FAO food security reports, a decrease in food security is taking place in the region. Since 2014, there has been an increase in the undernutrition prevalence of the region, the economic growth stopped, thus impacting the economy of the household and access to quality foods such as vegetables and fruits. This might have consequently decreased the intake of key micronutrients and increased the prevalence of overweight and obesity in the region (7).

With respect to the risks, our model showed an overall 12% increased risk for being obese in the lower SES compared with the high SES and 62% increased risk for being obese in women. It was also noted that the risk of being obese increases with age. This late finding raises an alarm since food insecurity gaps between the genders are wider in LA than the global gaps (7). This is important since we face the challenges of demographic changes for women of all the ages, including women of childbearing age, making women more vulnerable to this condition and its comorbidities and the emerge in food insecurity, a risk for obesity when the environment is deprived and poor (53). Food-insecure individuals have consistently been shown to consume less nutrient-dense healthy foods such as fruits, vegetables, and dairy products (54), while having an increased consumption of energy-dense foods such as high-fat dairy products, salty snacks, and sugar-sweetened beverages (55).

A limitation of this study is that it could not evaluate blood calcium nor iron deposit or ferritin status in the population studied, so we cannot compare intake vs. actual status of the micronutrients, which particularly for iron could have improved the evaluation of iron status. However, the assessment of intake used the Multiple-Pass method and was performed by very well-trained personnel in order to get the most accurate record of nutrient intake. The fact that two consecutive 24-h recalls were assessed and misreporting adjustments for evaluation of energy intake were made and minimized the errors often seen in the dietary recall of overweight individuals.

Hypothetically, we could think that because of the mechanisms of these micronutrients, promoting adequate intakes of calcium in a context such as the Latin American which is facing challenges to achieve food and nutritional security, adopting policies to promote a healthy diet, and fortify foods with micronutrients, when evidence shows the necessity to implement it, should be effective ways to contribute in the prevention of obesity and its associated comorbidities.

In addition, LA faces the challenge of the burden of cardiovascular diseases, type 2 diabetes, and dyslipidemias, which also affects the quality of life of the population and increases the cost of the investments in care of the affected population; therefore, a reduced prevalence of obesity would be expected to be followed by a reduced incidence and prevalence of all these diseases.

In 2016, a team of researchers from Mexico, Brazil, Chile, Colombia, Ecuador, Peru, Venezuela, Panama, and El Salvador examined the prevalence of cardiovascular disease including heart attack, heart failure, atrial fibrillation, and hypertension and this resulted in up to 6.8 million disability-adjusted life years or years of healthy life lost (56). These four cardiac disorders cost USD$30.9 billion in 2015 (16, 45). This is just an example of what can be the economic cost of cardiovascular diseases in Latin America, thus highlighting the relevance of formulating adequate policies to revert the risks through prevention on maintaining a healthy diet, a level of physical activity that combats sedentary lifestyles, and quality of the living conditions, all of which will have an impact in achieving healthier nutrition status for the population.

## Conclusion

This study shows that an important proportion of the adolescents and adults living in LA showed an alteration on its anthropometric profile with a tendency toward the overweight and obesity that coexist with an inadequate intake of calcium. This scenario predisposes the population to obesity and NCD and constitutes an economic load for the governments and societies in general. Thus, a comprehensive approach that influences the determinants of obesity, in each country, focusing on changing lifestyles in terms of prioritizing quality nutrition with nutrient-dense healthy foods, switching from physical inactivity, and sedentary patterns for stimulating physical activity is key to ensure well-being in the Latin American region.

## Data Availability Statement

The data analyzed in this study is subject to the following licenses/restrictions: the datasets presented in this article are not readily available because the data cannot be shared for privacy restrictions. Requests to access the datasets should be directed to Marianella Herrera-Cuenca, manyma@gmail.com.

## Ethics Statement

The studies involving human participants were reviewed and approved by Western Institutional Review Board (#20140605). Written informed consent to participate in this study was provided by the participants' legal guardian/next of kin.

## Author Contributions

All authors listed have made a substantial, direct and intellectual contribution to the work, and approved it for publication.

## Funding

The ELANS was initially supported by a scientific grant from the Coca Cola Company and support from the Ferrero, Instituto Pensi/Hospital Infantil Sabara, International Life Science Institute of Argentina, Universidad de Costa Rica, Pontificia Universidad Católica de Chile, Pontificia Universidad Javeriana, Universidad Central de Venezuela/Fundación Bengoa, Universidad San Francisco de Quito, and Instituto de Investigación Nutricional de Peru.

## Conflict of Interest

The authors declare that the research was conducted in the absence of any commercial or financial relationships that could be construed as a potential conflict of interest.

## Publisher's Note

All claims expressed in this article are solely those of the authors and do not necessarily represent those of their affiliated organizations, or those of the publisher, the editors and the reviewers. Any product that may be evaluated in this article, or claim that may be made by its manufacturer, is not guaranteed or endorsed by the publisher.

## ELANS Study Group

Chairs: Mauro Fisberg and Irina Kovalskys; co-chair: Georgina Gómez Salas; core group members: Attilio Rigotti, Lilia Yadira Cortés Sanabria, Martha Cecilia Yépez García, Rossina Gabriella Pareja Torres, and Marianella Herrera-Cuenca; steering committee: Berthold Koletzko, Luis A. Moreno, Michael Pratt, and Katherine L. Tucker; project managers: Viviana Guajardo and Ioná Zalcman Zimberg; International Life Sciences Institute, Argentina: Irina Kovalskys, Viviana Guajardo, María Paz Amigo, Ximena Janezic, and Fernando Cardini; Universidad I Salud: Myriam Echeverry and Martin Langsman; Instituto Pensi-Hospital Infantil Sabara, Brazil: Mauro Fisberg, Ioná Zalcman Zimberg, and Natasha Aparecida Grande de França; Pontificia Universidad Católica de Chile: Attilio Rigotti, Guadalupe Echeverría, Leslie Landaeta, and Óscar Castillo; Pontificia Universidad Javeriana, Colombia: Lilia Yadira Cortés Sanabria, Luz Nayibe Vargas, Luisa Fernanda Tobar, and Yuri Milena Castillo; Universidad de Costa Rica: Georgina Gómez Salas, Rafael Monge Rojas, and Anne Chinnock; Universidad San Francisco de Quito, Ecuador: Martha Cecilia Yépez García, Mónica Villar Cáceres, and María Belén Ocampo; Instituto de Investigación Nutricional, Perú: Rossina Pareja Torres, María Reyna Liria, Krysty Meza, Mellisa Abad, and Mary Penny; Universidad Central de Venezuela and Bengoa Foundation: Marianella Herrera-Cuenca, Maritza Landaeta-Jiménez, Betty Méndez-Pérez, Maura Vásquez, Omaira Rivas, Carmen Meza, Servando Ruiz, Guillermo Ramírez, and Pablo Hernández; accelerometry analysis: Priscila Bezerra Gonçalves and Claudia Alberico; and physical activity advisor: Gerson Luis de Moraes Ferrari.
