# Supplementary material for: Anthropometric Profile of Latin American Population: Results From the ELANS Study
Source: Front Nutr. 2021 Nov 8;8:740361. doi: 10.3389/fnut.2021.740361 (PMC8606788; doi:10.3389/fnut.2021.740361)
Supplement: Supplementary file 1 [file Data_Sheet_1.docx]

**Supplementary Table 1.** Demographic indicators of subjects in the Latin American Study of Nutrition and Health (ELANS) by country.

|  | Argentina | Brazil | Chile | Colombia | Costa Rica | Ecuador | Perú | Venezuela | ELANS |
| --- | --- | --- | --- | --- | --- | --- | --- | --- | --- |
|  | n (%) | n (%) | n (%) | n (%) | n (%) | n (%) | n (%) | n (%) | n (%) |
| **Gender** |  |  |  |  |  |  |  |  |  |
| Male | 406 (46.2) | 658 (46.8) | 306 (49.0) | 428 (48.9) | 277 (50.3) | 296 (51.6) | 428 (48.9) | 414 (48.0) | 3213 (48.3) |
| Female | 473 (53.8) | 748 (53.2) | 319 (51.0) | 447 (51.1) | 274 (49.7) | 278 (48.4) | 448 (51.1) | 448 (52.0) | 3435 (51.7) |
| **Age group** |  |  |  |  |  |  |  |  |  |
| 15-19 | 104 (11.8) | 158 (11.2) | 85 (13.6) | 86 (9.8) | 89 (16.2) | 86 (15.0) | 121 (13.8) | 118 (13.7) | 847 (12.7) |
| 20-34 | 294 (33.4) | 520 (37.0) | 214 (34.2) | 319 (36.5) | 210 (38.1) | 226 (39.4) | 357 (40.8) | 344 (39.9) | 2484 (37.4) |
| 35-49 | 267 (30.4) | 436 (31.0) | 175 (28.0) | 255 (29.1) | 147 (26.7) | 163 (28.4) | 243 (27.7) | 237 (27.5) | 1923 (28.9) |
| 50-65 | 214 (24.3) | 292 (20.8) | 151 (24.2) | 215 (24.6) | 105 (19.1) | 99 (17.2) | 155 (17.7) | 163 (18.9) | 1394 (21.0) |
| **SES** |  |  |  |  |  |  |  |  |  |
| High | 50 (5.7) | 128 (9.1) | 52 (8.3) | 49 (5.6) | 78 (14.2) | 73 (12.7) | 183 (20.9) | 43 (5.0) | 656 (9.9) |
| Middle | 402 (45.7) | 661 (47.0) | 283 (45.3) | 290 (33.1) | 300 (54.4) | 211 (36.8) | 277 (31.6) | 138 (16.0) | 2562 (38.5) |
| Low | 427 (48.6) | 617 (43.9) | 290 (46.4) | 536 (61.3) | 173 (31.4) | 290 (50.5) | 416 (47.5) | 681 (79.0) | 3430 (51.6) |
| **Total** | 879 (100.0) | 1406 (100.0) | 625 (100.0) | 875 (100.0) | 551 (100.0) | 574 (100.0) | 876 (100.0) | 862 (100.0) | 6648 (100.0) |

**Supplementary Table 2.** Association between country and obesity by gender in the Latin American Study of Nutrition and Health (ELANS).

| **Country** | **Gender** | OR | 95% CI | | p |
| --- | --- | --- | --- | --- | --- |
| Argentina | Male | 1.000 |  |  |  |
|  | Female | 1.496 | 1.110 | 2.016 | < 0.05 |
| Brazil | Male | 1.000 |  |  |  |
|  | Female | 1.509 | 1.183 | 1.926 | < 0.05 |
| Chile | Male | 1.000 |  |  |  |
|  | Female | 1.795 | 1.250 | 2.577 | < 0.05 |
| Colombia | Male | 1.000 |  |  |  |
|  | Female | 1.370 | 0.951 | 1.974 | > 0.05 |
| Costa Rica | Male | 1.000 |  |  |  |
|  | Female | 1.834 | 1.261 | 2.666 | < 0.05 |
| Ecuador | Male | 1.000 |  |  |  |
|  | Female | 2.076 | 1.408 | 3.061 | < 0.05 |
| Peru | Male | 1.000 |  |  |  |
|  | Female | 1.914 | 1.384 | 2.645 | < 0.05 |
| Venezuela | Male | 1.000 |  |  |  |
|  | Female | 1.505 | 1.119 | 2.024 | < 0.05 |

Abbreviations: OR, odds ratio; CI, confidence interval.

Regression model using Obesity (BMI) as dependent variable, adjusted for energy intake, age, socio-economic status and educational level.

**Supplementary Table 3.** Association between country and obesity by age group in the Latin American Study of Nutrition and Health (ELANS).

| **Country** | **Age group** | OR | 95% CI | | p |
| --- | --- | --- | --- | --- | --- |
| Argentina | 15-19 | 1.000 |  |  |  |
|  | 20-34 | 0.415 | 0.289 | 0.594 | < 0.05 |
|  | 35-49 | 1.424 | 1.043 | 1.946 | < 0.05 |
|  | 50-65 | 3.186 | 2.296 | 4.420 | < 0.05 |
| Brazil | 15-19 | 1.000 |  |  |  |
|  | 20-34 | 0.734 | 0.569 | 0.946 | < 0.05 |
|  | 35-49 | 1.351 | 1.048 | 1.740 | < 0.05 |
|  | 50-65 | 1.937 | 1.467 | 2.558 | < 0.05 |
| Chile | 15-19 | 1.000 |  |  |  |
|  | 20-34 | 0.578 | 0.39 | 0.855 | < 0.05 |
|  | 35-49 | 1.594 | 1.092 | 2.325 | < 0.05 |
|  | 50-65 | 1.704 | 1.153 | 2.519 | < 0.05 |
| Colombia | 15-19 | 1.000 |  |  |  |
|  | 20-34 | 0.603 | 0.432 | 0.842 | < 0.05 |
|  | 35-49 | 1.865 | 1.290 | 2.698 | < 0.05 |
|  | 50-65 | 1.603 | 1.076 | 2.390 | < 0.05 |
| Costa Rica | 15-19 | 1.000 |  |  |  |
|  | 20-34 | 0.514 | 0.341 | 0.773 | < 0.05 |
|  | 35-49 | 2.335 | 1.569 | 3.477 | < 0.05 |
|  | 50-65 | 2.178 | 1.398 | 3.392 | < 0.05 |
| Ecuador | 15-19 | 1.000 |  |  |  |
|  | 20-34 | 0.501 | 0.339 | 0.741 | < 0.05 |
|  | 35-49 | 1.897 | 1.269 | 2.834 | < 0.05 |
|  | 50-65 | 2.916 | 1.850 | 4.596 | < 0.05 |
| Peru | 15-19 | 1.000 |  |  |  |
|  | 20-34 | 0.474 | 0.312 | 0.720 | < 0.05 |
|  | 35-49 | 1.885 | 1.350 | 2.632 | < 0.05 |
|  | 50-65 | 2.070 | 1.420 | 3.017 | < 0.05 |
| Venezuela | 15-19 | 1.000 |  |  |  |
|  | 20-34 | 0.461 | 0.333 | 0.639 | < 0.05 |
|  | 35-49 | 2.197 | 1.607 | 3.003 | < 0.05 |
|  | 50-65 | 1.954 | 1.381 | 2.765 | < 0.05 |

Abbreviations: OR, odds ratio; CI, confidence interval.

Regression model using Obesity (BMI) as dependent variable, adjusted for energy intake, sex, socio-economic status and educational level.

**Supplementary Table 4.** Association between country and obesity by Socio-economic status in the Latin American Study of Nutrition and Health (ELANS).

| **Country** | **Socio-economic status** | OR | 95% CI | | p |
| --- | --- | --- | --- | --- | --- |
| Argentina | High | 1.000 |  |  |  |
|  | Middle | 0.838 | 0.622 | 1.13 | > 0.05 |
|  | Low | 1.435 | 1.066 | 1.932 | < 0.05 |
| Brazil | High | 1.000 |  |  |  |
|  | Middle | 0.896 | 0.704 | 1.14 | > 0.05 |
|  | Low | 1.042 | 0.818 | 1.327 | > 0.05 |
| Chile | High | 1.000 |  |  |  |
|  | Middle | 0.727 | 0.503 | 1.051 | > 0.05 |
|  | Low | 1.799 | 1.258 | 2.572 | < 0.05 |
| Colombia | High | 1.000 |  |  |  |
|  | Middle | 0.92 | 0.641 | 1.323 | > 0.05 |
|  | Low | 1.235 | 0.860 | 1.773 | > 0.05 |
| Costa Rica | High | 1.000 |  |  |  |
|  | Middle | 0.639 | 0.443 | 0.922 | < 0.05 |
|  | Low | 1.148 | 0.768 | 1.717 | > 0.05 |
| Ecuador | High | 1.000 |  |  |  |
|  | Middle | 0.775 | 0.519 | 1.158 | > 0.05 |
|  | Low | 1.313 | 0.897 | 1.923 | > 0.05 |
| Peru | High | 1.000 |  |  |  |
|  | Middle | 1.267 | 0.91 | 1.765 | > 0.05 |
|  | Low | 0.714 | 0.519 | 0.982 | < 0.05 |
| Venezuela | High | 1.000 |  |  |  |
|  | Middle | 1.158 | 0.78 | 1.721 | > 0.05 |
|  | Low | 0.793 | 0.557 | 1.129 | > 0.05 |

Abbreviations: OR, odds ratio; CI, confidence interval.

Regression model using Obesity (BMI) as dependent variable, adjusted for energy intake, age, age and educational level.

**Supplementary Table 5.** Association between country and obesity by intake of micronutrients in the Latin American Study of Nutrition and Health (ELANS).

| **Country** | **Intake** | OR | 95% CI | | p |
| --- | --- | --- | --- | --- | --- |
| Argentina | Within the iron recommendation | 1.000 |  |  |  |
|  | Under iron recommendation | 0.370 | 0.045 | 3.022 | > 0.05 |
| Brazil | Within the iron recommendation | 1.000 |  |  |  |
|  | Under iron recommendation | 0.764 | 0.538 | 1.084 | > 0.05 |
| Chile | Within the iron recommendation | 1.000 |  |  |  |
|  | Under iron recommendation | 0.571 | 0.122 | 2.670 | > 0.05 |
| Colombia | Within the iron recommendation | 1.000 |  |  |  |
|  | Under iron recommendation | - | - | - | - |
| Costa Rica | Within the iron recommendation | 1.000 |  |  |  |
|  | Under iron recommendation | 0.674 | 0.138 | 3.278 | > 0.05 |
| Ecuador | Within the iron recommendation | 1.000 |  |  |  |
|  | Under iron recommendation | - | - | - | - |
| Peru | Within the iron recommendation | 1.000 |  |  |  |
|  | Under iron recommendation | 3.372 | 0.675 | 16.837 | > 0.05 |
| Venezuela | Within the iron recommendation | 1.000 |  |  |  |
|  | Under iron recommendation | 2.824 | 1.013 | 7.873 | < 0.05 |
| Argentina | Within the calcium recommendation | 1.000 |  |  |  |
|  | Under calcium recommendation | 1.790 | 1.274 | 2.517 | < 0.05 |
| Brazil | Within the calcium recommendation | 1.000 |  |  |  |
|  | Under calcium recommendation | 0.753 | 0.463 | 1.225 | > 0.05 |
| Chile | Within the calcium recommendation | 1.000 |  |  |  |
|  | Under calcium recommendation | 0.672 | 0.340 | 1.326 | > 0.05 |
| Colombia | Within the calcium recommendation | 1.000 |  |  |  |
|  | Under calcium recommendation | 1.207 | 0.780 | 1.868 | > 0.05 |
| Costa Rica | Within the calcium recommendation | 1.000 |  |  |  |
|  | Under calcium recommendation | 1.493 | 0.484 | 4.607 | > 0.05 |
| Ecuador | Within the calcium recommendation | 1.000 |  |  |  |
|  | Under calcium recommendation | 1.158 | 0.696 | 1.926 | > 0.05 |
| Peru | Within the calcium recommendation | 1.000 |  |  |  |
|  | Under calcium recommendation | 0.897 | 0.286 | 2.813 | > 0.05 |
| Venezuela | Within the calcium recommendation | 1.000 |  |  |  |
|  | Under calcium recommendation | 1.076 | 0.748 | 1.546 | > 0.05 |

Abbreviations: OR, odds ratio; CI, confidence interval.

Regression model using Obesity (BMI) as dependent variable, adjusted for energy intake, sex, age, socio-economic status and educational level.
